# Abstract of Meteorological Observations for November, 1855, Made at Philadelphia, Pa.

**Published:** 1856-01

**Authors:** James A. Kirkpatrick


*Abstract of Meteorological Observations for November,* 1855, *made at Philadelphia, Pa. Latitude* 39° 57′ 28″ *N., Longitude* 75° 10′ 40″ *W. from Greenwich.* By Prof. James A. Kirkpatrick.

| 1855. November. | Barometer. Daily Mean | Barometer. Mean Daily Range. | Thermom. Daily Mean | Thermom. Mean Daily Range | Dew Point 2 P.M. | Force of Vapor 2 P.M. | Rel. Humid. 2 P.M. | Rain. | Prevailing Winds. | Remarks. |
|---|---|---|---|---|---|---|---|---|---|---|
| | Inches. | Inches. | Deg. | Deg. | Deg. | Inches. | Hunds. | Inch. | Points. | |
| 1 | 29.866 | .291 | 59.3 | 6.0 | 57.8 | .479 | .83 | | NE. | M. fog, ev. drizzling. |
| 2 | 29.862 | .101 | 60.0 | 2.0 | 57.0 | .466 | .78 | | NNE. | Cloudy; ev. *rain.* |
| 3 | 29.933 | .097 | 58.5 | 2.5 | 58.1 | .484 | .97 | 0.918 | E. | *Raining* all day. |
| 4 | 30.048 | .115 | 53.8 | 4.7 | 51.4 | .380 | .76 | | NE. | Cl'dy; light drizzling *rain* all day. |
| 5 | 30.199 | .152 | 49.5 | 4.2 | 41.0 | .257 | .56 | | NE. | Cloudy. |
| 6 | 30.101 | .098 | 50.5 | 1.7 | 43.4 | .282 | .67 | | NE. | Cloudy. |
| 7 | 29.996 | .105 | 54.3 | 3.8 | 49.4 | .352 | .70 | | (Var.) | Cloudy. |
| 8 | 29.971 | .051 | 55.3 | 2.3 | 53.6 | .412 | .77 | | NE. | M. fog, cloudy. |
| 9 | 30.202 | .231 | 51.3 | 4.0 | 36.5 | .216 | .43 | | N. | Clear. |
| 10 | 30.173 | .048 | 51.8 | 2.5 | 43.5 | .283 | .56 | | NE. | Cl'dy. *Bar. highest* 30.228. |
| 11 | 30.078 | .095 | 56.8 | 5.0 | 51.1 | .375 | .59 | | NE. | M. fog, cloudy. |
| 12 | 29.971 | .106 | 55.2 | 3.7 | 54.2 | .420 | .94 | 0.333 | NE. | Cloudy; ev. add night *rain.* |
| 13 | 30.022 | .118 | 58.2 | 3.3 | 43.6 | .284 | .51 | | NW. | M. cloudy; aft. & ev. clear. |
| 14 | 3 .077 | .073 | 50.7 | 5.2 | 42.3 | .270 | .50 | | NW. | Clear. |
| 15 | 30.036 | .041 | 54.3 | 3.7 | 41.0 | .257 | .43 | | (Var.) | Clear. |
| 16 | 29.758 | .278 | 57.7 | 3.3 | 54.6 | .426 | .64 | | SW. | Cloudy. *Therm. highest* 67° |
| 17 | 29.931 | .176 | 42.3 | 15.3 | 28.3 | .155 | .57 | 0.122 | NE. | Cloudy; night *rain.* |
| 18 | 29.969 | .123 | 43.5 | 2.5 | 28.9 | .159 | .46 | | NW. | Aft. cloudy; m. & ev. clear. |
| 19 | 30.057 | .089 | 40.0 | 3.5 | 24.7 | .133 | .42 | | W. | lear. |
| 20 | 30.208 | .150 | 36.3 | 3.7 | 20.5 | .110 | .46 | | NW. | M. & aft. cloudy; ev. clear. |
| 21 | 29.864 | .344 | 40.8 | 4.5 | 41.8 | .265 | .91 | 0.593 | (Var.) | Cloudy; m. *snow* 7 to 8, then *rain* to 5 P.M |
| 22 | 30.108 | .287 | 36.0 | 4.8 | 23.0 | .123 | .54 | | NW. | M. cl'dy, aft. and ev. clear. |
| 23 | 29.839 | .360 | 38.7 | 6.3 | 31.0 | .173 | .60 | | SW. | Cloudy. *Therm. lowest* 28° |
| 24 | 29.942 | .322 | 41.8 | 6.8 | 14.6 | .084 | .28 | | NW. | M. cl'dy; aft. and ev. clear. |
| 25 | 29.864 | .284 | 44.5 | 10.0 | 43.6 | .284 | .78 | 0.056 | (Var.) | Cloudy; aft. *rain.* |
| 26 | 29.617 | .285 | 51.2 | 12.3 | 40.9 | .256 | .49 | | SW. | M. and aft. cl'dy; ev. clear. |
| 27 | 29.715 | .194 | 42.7 | 8.5 | 29.6 | .164 | ,46 | | SW. | Clear; |
| 28 | 29.386 | .329 | 43.5 | 0.8 | 35.8 | .210 | .58 | | SW. | Cloudy; *Bar. lowest* 29.330. |
| 29 | 29.637 | .278 | 33.2 | 10.3 | 13.2 | .079 | .44 | | WNW. | M. cl'dy; aft. and ev. clear. |
| 30 | 30.014 | .376 | 36.7 | 8.7 | 27.7 | .151 | .52 | | (Var.) | Clear. |
| Means for Nov., 1855 | 29.948 | .187 | 48.3 | 5.2 | 39.4 | .266 | .60 | 2.022 | N. 64° 5′ W. 43-100. | |
| Means for Nov., 5 yrs. | 29.954 | .187 | 45.3 | 5.5 | 37.7 | | .60 | 3.463 | N. 57° 35′ W. 41-100. | |
| Means for Aut'n. 1855 | 29.920 | .151 | 57.9 | 5.5 | 49.0 | .388 | .61 | 9.567 | N. 67° 53′ W. 38-100. | |
| Means for Aut'n. 5 yrs. | 29.965 | .151 | 56.8 | 5.5 | 48.4 | | 61. | 9.917 | N. 73° 50′ W. 42-100. | |

The Monthly Range of the Mercury in the Barometer was 0.898 of an inch, and in the Thermometer 39°.

Correction.—In the Abstract for October, vol. xi., p. 762, the Mean Temperature of the month for 1855 should have been printed 55.2°, and the Mean Temperature of the month for five years, 56 7°.

